# The Importance of Persistence

**DOI:** 10.1093/cid/ciw525

**Published:** 2016-11-02

**Authors:** Stanley A. Plotkin

**Affiliations:** University of Pennsylvania and Vaxconsult, Doylestown

**Keywords:** pertussis, waning immunity, Th1 response, Th17 response

“Energy and persistence conquer all things.” — Benjamin Franklin

It is amazing that in the 21st century, more than 100 years since Jules Bordet discovered the organism that bears his name, *Bordetella pertussis*, we are far from a full understanding of the organism, the disease, the correlates of protection, and the ideal vaccine. Unlike many infectious diseases, natural infection does not give lifelong immunity, and unlike our best vaccines, immunity after vaccination is more or less transient. Therefore, it is not surprising that control of pertussis is relatively poor, although generalizations are difficult because of geographical differences.

The whole-cell pertussis vaccine that evolved in the 20th century was clearly reactogenic, although the permanence of its sequelae was exaggerated. Nevertheless, reactions were disturbing to parents and, even worse, whole-cell vaccines were extremely variable in the protection they gave, as was starkly evident in the results of the trials conducted in the 1990s, in which whole-cell vaccines were used as comparators to acellular vaccines. Specifically, whole-cell vaccines made in some European countries were shown to be much more efficacious than one made in the United States. The efficacy of vaccines made by laboratories in other parts of the world remains uncertain.

The reactions to whole-cell vaccines in high-income countries, including convulsions and hypotonic episodes, led to its rejection and replacement by acellular vaccines composed of 1–5 virulence factors of the organism. The fact that the composition of acellular vaccines has been so variable, both in the number of antigens and their concentration, testifies to the uncertainty about which factors are important, an ignorance that continues today to a certain extent. However, there was general agreement that pertussis toxin (PT), with its multiple systemic and local effects, should be included as toxoid. There was disagreement as to the need to add filamentous hemagglutinin (FHA), pertactin, and fimbria.

The situation today is that in many middle-income countries, notably in Latin America, whole-cell vaccines made by a variety of manufacturers are widely used with varying and uncertain evidence for safety and efficacy. However, in most high-income countries, including the United States, Australia, and Western Europe, acellular vaccines are the rule, but waning immunity has resulted in a resurgence of pertussis. Moreover, the tendency of low- and middle-income countries to switch to acellular pertussis vaccines has stopped in its tracks pending improvements in them. And yet, as this supplement shows, pertussis remains a problem regardless of the economic level of a country. If immunity to the infection is impermanent even after natural infection, it is even less so after vaccination. The result is the continued circulation of the bacterium in family contacts, regardless of their vaccination history, resulting in exposure of vulnerable newborns.

So what is the way forward? I submit that one of the first steps, perhaps under the aegis of the World Health Organization, is to examine all the whole-cell vaccines being used in the world today for evidence of safety and immunogenicity, both with regard to antibody responses to the known protective antigens such as PT, pertactin, and fimbrial agglutinogens and also T-cell orientation: Th1, Th2, and Th17. These results could allow the elimination of weakly immunogenic and strongly reactogenic whole-cell vaccines in favor of better ones.

The second step, not surprisingly, consists of improvements in acellular vaccines. A key study would be to determine whether the results in baboons suggesting that acellular vaccines do not prevent carriage of *Bordetella pertussis* and therefore do not confer herd immunity can be confirmed or contradicted in humans. It may be that the acellular vaccines reduce but do not eliminate nasopharyngeal carriage, but even this information would be critical.

Beyond that, there are some steps that seem obvious. First is the replacement of chemically inactivated PT with genetically inactivated PT. This would avoid the destruction of epitopes that induce bactericidal antibodies and increase the immune response to PT, perhaps prolonging it so that waning immunity would be less pronounced. Second, various adjuvants that give stronger immune stimuli than aluminum compounds should be tested to determine whether a more Th1/Th17 orientation would prolong protection by acellular vaccines. Now that oil-in-water adjuvants and Toll-like receptor agonists have been used in other vaccines, it is not impossible to consider going beyond alum.

An important question in this regard is whether children who received acellular vaccines in infancy can have their immune systems reoriented by an adjuvanted vaccine. However, as it appears that even 1 dose of whole-cell vaccine in infancy orients pertussis immune responses toward later persistent protection, reorientation of the immune response by an adjuvanted vaccine may not be possible.

What other components should be added to new acellular vaccines? The increased prevalence of pertactin-negative strains argue against the inclusion of this antigen in future vaccines. FHA appears to provide some improvement in protection by PT vaccines, and the evidence for the importance of fimbrial agglutinogen 2 is fairly solid. Beyond that, one enters into unknown territory and regulatory uncertainty. Adenylate cyclase is the most obvious choice for an additional antigen, but there are many other virulence factors that have been proposed. Perhaps most intriguing is the idea of using an attenuated *B. pertussis* strain as a booster of nasopharyngeal and systemic immunity against pertussis. The latter possibility should be urgently explored.

However, it should be remembered that the current acellular pertussis vaccines have not been complete failures. In fact, they do give strong immediate protection of vaccinated children against serious disease. Their problem is to achieve persistent memory that prolongs the protection beyond 2–3 years. To solve this deficiency, we must learn more about B-cell memory and about how to orient T-cell responses to aid persistence of protection. In addition, those countries that continue to use whole-cell vaccines must be certain that the ones they use are as immunogenic and safe as possible. This supplement is a step in the direction of resolving problems with one of the oldest vaccines still in use, as well as its modern avatar that turned out not to be as good as we hoped.

